# Tagging Single Nucleotide Polymorphisms in the *BRIP1* Gene and Susceptibility to Breast and Ovarian Cancer

**DOI:** 10.1371/journal.pone.0000268

**Published:** 2007-03-07

**Authors:** Honglin Song, Susan J. Ramus, Susanne Krüger Kjaer, Estrid Hogdall, Richard A. DiCioccio, Alice S. Whittemore, Valerie McGuire, Claus Hogdall, Ian J. Jacobs, Douglas F. Easton, Bruce A.J. Ponder, Alison M. Dunning, Simon A. Gayther, Paul D.P. Pharoah

**Affiliations:** 1 Cancer Research UK (CRUK) Department of Oncology, University of Cambridge, Strangeways Research Laboratory, Cambridge, United Kingdom; 2 Translational Research Laboratories, Institute for Women's Health, University College London, United Kingdom; 3 Institute of Cancer Epidemiology, Danish Cancer Society, Copenhagen, Denmark; 4 Department of Cancer Genetics, Roswell Park Cancer Institute, Buffalo, New York, United States of America; 5 Department of Health Research and Policy, Stanford University School of Medicine, Stanford, United States of America; 6 The Juliane Marie Centre, Rigshospitalet, University of Copenhagen, Copenhagen, Denmark; 7 Cancer Research UK (CRUK) Genetic Epidemiology Unit, University of Cambridge, Strangeways Research Laboratory, Cambridge, United Kingdom; Ordway Research Institute, Inc., United States of America

## Abstract

**Background:**

*BRIP1* interacts with *BRCA1* and functions in regulating DNA double strand break repair pathways. Germline *BRIP1* mutations are associated with breast cancer and Fanconi anemia. Thus, common variants in the *BRIP1* are candidates for breast and ovarian cancer susceptibility.

**Methods:**

We used a SNP tagging approach to evaluate the association between common variants (minor allele frequency≥0.05) in *BRIP1* and the risks of breast cancer and invasive ovarian cancer. 12 tagging SNPs (tSNPs) in the gene were identified and genotyped in up to 2,270 breast cancer cases and 2,280 controls from the UK and up to 1,513 invasive ovarian cancer cases and 2,515 controls from the UK, Denmark and USA. Genotype frequencies in cases and controls were compared using logistic regression.

**Results:**

Two tSNPs showed a marginal significant association with ovarian cancer: Carriers of the minor allele of rs2191249 were at reduced risk compared with the common homozygotes (Odds Ratio (OR) = 0.90 (95% CI, 0.82–1.0), P-trend = 0.045) and the minor allele of rs4988344 was associated with increased risk (OR = 1.15 (95%CI, 1.02–1.30), P-trend = 0.02). When the analyses were restricted to serous ovarian cancers, these effects became slightly stronger. These results were not significant at the 5% level after adjusting for multiple testing. None of the tSNPs was associated with breast cancer.

**Conclusions:**

It is unlikely that common variants in *BRIP1* contribute significantly to breast cancer susceptibility. The possible association of rs2191249 and rs4988344 with ovarian cancer risks warrant confirmation in independent case-control studies.

## Introduction

Breast and ovarian cancer are among the most frequent cancers in women in Western countries [1], [2]. Combined, there were approximately 49,000 cases of breast and ovarian cancer reported in the United Kingdom in 2002 and more than 17,000 deaths from these cancers in 2003. The main breast/ovarian cancer predisposition genes so far identified are *BRCA1* and *BRCA2*. They are estimated to account for <40% of the excess familial risk of ovarian cancer and <25% of the excess familial breast cancer risk [3], [4]. Thus, other breast/ovarian cancer susceptibility genes are likely to exist. Linkage studies in non-*BRCA1/2* breast cancer families and segregation analysis in ovarian cancer families suggest that a large fraction of the residual familial risks is due to multiple alleles of moderate to low penetrance rather than additional high penetrance susceptibility genes [5], [6].

It is widely accepted that tumour formation is a multistep process accompanied by an accumulation of multiple genetic alterations. Tumour development is associated with a breakdown in the mechanisms that control cell division and the maintenance of genome integrity (e.g. DNA repair). Thus, genes involved in these pathways represent good candidates for genetic susceptibility. *BRIP1* (*BRCA1*-interacting protein 1), also called *BACH1* (*BRCA1*-associated C-terminal helicase-1) and *FANCJ* (Fanconi anemia complementation group J), belongs to the DEAH helicase family [7], [8]. It works as both a DNA-dependent ATPase and a 5-prime-to-3 prime DNA helicase, and is essential for DNA repair and genomic stability [9]. It is universally expressed and co-localised with *BRCA1* in nuclear foci [7]. The complex formed by *BRIP1* and the BRCT repeats of *BRCA1* is important for the role of *BRCA1* in its DNA double strand break repair and tumour suppressor functions [7]. This specific interaction between *BRCA1* and phosphorylated *BRIP1* is regulated by the cell cycle and is essential for DNA damage-induced checkpoint control during G2 to M phase transition [10]. Germline mutations in *BRIP1* are associated with Fanconi anemia, which is a chromosome instability disorder characterized by developmental abnormalities, bone marrow failure and predisposition to cancer [8], [11]. Very recently, a rare ptotein truncating variant in *BRIP1* has been identified as low-penetrance breast cancer susceptibility alleles [12].

The *BRIP1* gene spans 180 kb, comprises 20 exons and encodes a protein of 1,249 amino acids [7]. It is located on chromosome 17q22, just distal to the *BRCA1* gene located at 17q21 [13], a region that is frequently somatically altered in both breast and ovarian cancer [14]–[15].

Taken together, these data suggest that *BRIP1* is a good candidate for moderate/low penetrance genetic susceptibility to breast and ovarian cancer. Several studies have already attempted to evaluate the association of individual *BRIP1* variants with breast cancer risk either by mutation analysis or genotyping a few SNPs within the gene [16]–[21], but only one included more than 1000 cases and none comprehensively evaluated all common variation in the gene. The aim of this study was to evaluate whether common variants in *BRIP1* are associated with breast and ovarian cancer risks using a SNP tagging approach within an association study design.

## Materials and Methods

### Breast cancer study subjects

Cases were drawn from SEARCH (breast cancer), an ongoing population based study, with cases ascertained through the East Anglian Cancer Registry. All patients diagnosed with invasive breast cancer below age 55 years since 1991 and still alive in 1996 (prevalent cases, median age 48 years), together with all those diagnosed <70 years between 1996 and the present (incident cases, median age 54 years) were eligible to take part. Sixty seven percent of eligible breast cancer patients returned a questionnaire and 64% provided a blood sample for DNA analysis. Controls were randomly selected from the Norfolk component of EPIC (European Prospective Investigation of Cancer) and somewhat older than cases (median age 63 years) though from a broadly similar age range. The ethnic background of cases and controls was established from the questionnaire- more than 98% of all subjects were white. The study has been approved by the Eastern Region Multicentre Research Ethics Committee, and all patients gave written informed consent. In total, 4473 cases and 4560 controls were available for analysis.

The samples were split into two sets in order to save DNA and reduce genotyping costs: the first set (n = 2270 cases and 2280 controls) was genotyped for all SNPs and the second set (n = 2203 cases and 2280 controls) were then tested for those SNPs that showed marginally significant associations in set 1 (P-heterogeneity or P-trend<0.1). This staged approach substantially reduces genotyping costs without significantly affecting statistical power. Cases were randomly selected for set 1 from the first 3,500 recruited, with set 2 comprising the remainder of these plus the next 974 incident cases recruited. As the prevalent cases were recruited first, the proportion of prevalent cases was somewhat higher in set 1 than set 2 (33% vs 20%). Median age at diagnosis is similar in both sets (51 and 52 years old respectively). There was no significant difference in the morphology, histopathological grade or clinical stage of the cases by set or by prevalent/incident status.

### Ovarian cancer study subjects

Ovarian cancer cases and controls from three different studies were used: the SEARCH ovarian cancer study from the United Kingdom, the MALOVA study from Denmark, and the FROC study from the USA. We have described these studies in detail in a previous report [22]. Briefly, the SEARCH ovarian cancer study comprises 730 invasive epithelial ovarian cancer cases collected from the East Anglian, West Midlands cancer registries in the UK. The same series of 2280 subjects from the EPIC-Norfolk cohort described for the breast cancer study (see above) were used as controls for the ovarian cancer study, plus a further 855 randomly selected female controls from the same cohort. The MALOVA study comprises 456 invasive epithelial ovarian cancer cases (median age 60 years) and 1231 controls (median age 57 years) randomly drawn from the same study area. Controls were of similar age range as cases, but not individually age matched. The FROC study contains 327 invasive ovarian cases and 429 age-matched controls – non-white subjects from FROC were excluded from this analysis.

### Tag SNP selection

The principal hypothesis underlying the experiment is that there are one or more common SNPs in *BRIP1* that are associated with an altered risk of the ovarian/breast cancer. We therefore aimed to identify a set of tagging SNPs [tSNPs] that efficiently tag all the known common variants (MAF>0.05) and are likely to tag most of the unknown common variants. We used data from the International HapMap Project [Phase II #20] to select tSNPs. The HapMap Project has genotyped a large number of SNPs in several populations. We used data from the 30 parent-offspring trios collected in 1980 from U.S. residents with North-Western European ancestry by the Centre d'Etude du Polymorphisme Humain [CEPH] for tSNP selection. The programme Tagger uses a strategy that combines the simplicity of pairwise methods with the potential efficiency of multimarker approaches [http://www.broad.mit.edu/mpg/tagger/] [23]. The best measure of the extent to which one SNP tags another SNP is the pairwise correlation coefficient [r_p_^2^], since the loss in power incurred by using a marker SNP in place of a true causal SNP is directly related to this measure. We aimed to define a set of tagging SNPs such that all known common SNPs had an estimated r_p_^2^>0.8 with at least one tagging SNP. However, some SNPs are poorly correlated with other single SNPs but may be efficiently tagged by a haplotype defined by multiple SNPs, thus reducing the number of tagging SNPs needed. As an alternative, therefore, we aimed for the correlation between each SNP and a haplotype of tagging SNPs [r_s_^2^] to be at>0.8.

### Genotyping

All samples were genotyped using the Taqman™ 7900HT Sequence Detection System according to manufacturer's instructions. Each assay was carried out using 10ng DNA in a 5 µl reaction using TaqMan universal PCR master mix (Applied Biosystems), forward and reverse primers and FAM and VIC labelled probes designed by Applied Biosystems (ABI Assay-by-design). Primer and probe sequences and assay conditions used for each polymorphism analysed are detailed in Table S1. All assays were carried out in 384-well plates and included 12 duplicate samples in each plate for quality control. Genotypes were determined using Allelic Discrimination Sequence Detection Software (Applied Biosystems, Warrington UK). DNA samples that did not give a clear genotype at the first attempt were not repeated. The criteria for a successful genotype is that the concordance rate for the duplicates should be ≥98% and the overall call rate>95% (by study) with >90% for any individual 384-well plate. Plates failing this should be excluded. Hence, there are variations in the number of samples successfully genotyped for each polymorphism.

### Statistics

Deviations of genotype frequencies in controls from those expected under Hardy-Weinberg equilibrium (HWE) were assessed by χ^2^ tests (1 d.f). The primary tests of association were univariate analyses between each tagging SNP and breast/ovarian cancer. Genotype frequencies were compared in cases and controls using unconditional logistic regression. Genotype specific risks with the common homozygote as the baseline comparator were estimated as odds ratios (OR) with associated 95% confidence intervals (95% CI) by unconditional logistic regression. We also tested for rare allele dose effect (assuming a multiplicative model). For the ovarian cancer data, all the analyses were stratified by study. We tested for heterogeneity between study strata by comparing logistic regression models with and without a genotype-stratum interaction term using likelihood-ratio tests.

In addition to the univariate analyses we carried out specific haplotype tests for those combinations of alleles that tagged specific SNPs. We also carried out a general comparison of common haplotype frequencies in each gene haplotype block utilising the data from all the tSNPs in that block. Haplotype blocks were defined such that the common haplotypes (>5% frequency) accounted for at least 80% of the haplotype diversity. We considered haplotypes with greater than 2% frequency in at least one study to be "common". Rare haplotypes were pooled. For both specific haplotype marker tests and the general comparison of haplotype frequencies by haplotype block, haplotype frequencies and subject-specific expected haplotype indicators were calculated separately for each study using the programme TagSNPs [24]. This implements an expectation substitution approach to account for haplotype uncertainty given unphased genotype data. Subjects missing >50% genotype data in each block were excluded from haplotype analysis. We used unconditional logistic regression to test the null hypothesis of no association between specific multi-marker tagging haplotype and cancer, by comparing a model with terms for subject specific-haplotype indicator with the intercept only model. The global null hypothesis of no association between haplotype frequency (by haplotype block) and cancer was tested, by comparing a model with multiplicative effects for each common haplotype (treating the most common haplotype as the referent) to the intercept-only model. Haplotype specific odds ratios were also estimated with their associated confidence intervals [25].

## Results

Using Hapmap CEPH data (Phase II #20), we identified 92 common SNPs in 198kb region containing the *BRIP1* gene (including 9kb up stream and 9 kb down stream of the gene). Sixteen SNPs were initially chosen as tagging SNPs (tSNPs), but four assays failed design. Thus, 12 tSNPs were selected for genotyping. The mean r_p_^2^ was 0.93 with 87 out of 92 SNPs tagged by a single marker with r_p_^2^>0.8. Two SNPs were tagged with multi-marker haplotypes with r_s_^2^>0.8 and one SNP was tagged with r^2^ = 0.78. The two SNPs for which the assays failed were poorly correlated with any other SNPs and so alternative tSNPs could not be selected. These two were tagged with r_p_^2^ of 0.16 and 0.06, respectively.

The 12 tSNPs were genotyped in approximately 2,300 breast cancer cases and a similar number of controls from the UK. For 11 tSNPs, genotype frequency distributions in controls were close to those expected under Hardy Weinberg Equilibrium (HWE) (table S2). Genotype calling for one tSNP (rs6504074) did not pass the quality assessment criteria with genotyping call rate <95%. Thus, this tSNP was removed from further analyses. There was no difference in genotype frequencies between incident and prevalent cases, nor was time between diagnosis and entry into the study associated with genotype (data not show). The observed genotype frequencies in breast cancer subjects are presented in Table S2. Table 1 shows the results of the test for the comparison of genotype frequencies (P-heterogeneity) between cases and controls. There were no significant differences in genotype frequencies between cases and controls for any of the 11 tSNPs tested (P>0.1), and no SNP was genotyped in the second set of samples. Genotype specific risks were close to unity and the tests for trend were not significant for any tSNPs.

**Table 1:** Breast and ovarian cancer genotype specific risks for each tSNP by study

*dbSNP*	Study	# cases	# controls	HetOR*[95% CI]	HomOR*[95% CI]	P_het	P_trend
rs11871785	**Breast Cancer**	2181	2267	0.97 [0.85–1.09]	1.11 [0.91–1.34]	0.39	0.61
	**Ovarian Cancer**	1492	4750	1.07 [0.95–1.22]	1.05 [0.86–1.27]	0.55	0.41
rs1557720	**Breast Cancer**	2156	2247	0.97 [0.85–1.10]	0.93 [0.78–1.10]	0.69	0.39
	**Ovarian Cancer**	1332	4229	1.05 [0.92–1.21]	1.08 [0.90–1.31]	0.65	0.36
rs11652980	**Breast Cancer**	2182	2275	0.97 [0.80–1.16]	0.69 [0.19–2.46]	0.80	0.62
	**Ovarian Cancer**	1490	4765	0.95 [0.78–1.17]	1.21 [0.38–3.91]	0.85	0.73
rs2191249	**Breast Cancer**	2189	2277	0.97 [0.86–1.10]	1.14 [0.90–1.43]	0.42	0.64
	**Ovarian Cancer**	1328	4242	0.93 [0.82–1.07]	**0.75 [0.58–0.99]**	0.09	**0.045**
rs16945628	**Breast Cancer**	2176	2271	0.97 [0.85–1.09]	1.09 [0.90–1.32]	0.48	0.70
	**Ovarian Cancer**	1495	4754	0.92 [0.81–1.05]	0.89 [0.73–1.09]	0.33	0.15
rs2191248	**Breast Cancer**	2162	2264	1.05 [0.93–1.20]	1.02 [0.84–1.23]	0.71	0.62
	**Ovarian Cancer**	1483	4741	1.02 [0.90–1.16]	1.12 [0.92–1.35]	0.51	0.32
rs16945643	**Breast Cancer**	2171	2265	1.02 [0.85–1.21]	1.05 [0.51–2.15]	0.98	0.82
	**Ovarian Cancer**	1485	4746	1.01 [0.86–1.20]	1.20 [0.58–2.49]	0.89	0.75
rs6504074	**Breast Cancer**	ND	ND	ND	ND	ND	ND
	**Ovarian Cancer**	1309	3968	0.94 [0.81–1.10]	0.85 [0.64–1.12]	0.26	0.25
rs2378908	**Breast Cancer**	2190	2277	1.04 [0.90–1.20]	0.74 [0.47–1.17]	0.36	0.86
	**Ovarian Cancer**	1302	4254	1.15 [0.99–1.33]	1.11 [0.70–1.77]	0.20	0.09
rs4988344	**Breast Cancer**	2189	2278	0.98 [0.86–1.12]	1.02 [0.69–1.50]	0.97	0.89
	**Ovarian Cancer**	1330	4265	1.11 [0.97–1.28]	**1.49 [1.03–2.16]**	0.05	**0.02**
rs9908659	**Breast Cancer**	2164	2266	0.97 [0.86–1.10]	1.04 [0.87–1.24]	0.74	0.85
	**Ovarian Cancer**	1492	4749	0.99 [0.87–1.13]	1.06 [0.88–1.27]	0.78	0.64
rs2048718	**Breast Cancer**	2170	2264	0.92 [0.81–1.06]	0.99 [0.83–1.17]	0.46	0.76
	**Ovarian Cancer**	1473	4741	0.93 [0.81–1.06]	1.01 [0.86–1.20]	0.39	0.98

1 odds ratio, 2 confidence interval, * compared with common homozygote. Confidence intervals that do not reach or cross 1.00 and P- values<0.05 are in bold type

One SNP (rs4986765) was tagged by a haplotype comprising the three common alleles of the tSNPs rs2191249, rs11871785 and rs16945628. The frequency of this haplotype was similar in cases (0.33) and in controls (0.32) (P = 0.33). Another SNP (rs1243935) was tagged by the haplotype comprising two rare alleles of tSNPs rs2191249 and rs6504074, but as the assay for the second of these SNPs failed the specific haplotype frequency in cases and controls could not be compared.

Genotyping quality was satisfactory for all 12 tSNPs in the ovarian cancer studies. Genotype frequency distributions in controls were close to those expected under HWE for all tSNPs (table S3) - rs2191248 deviated slightly from HWE in one of the three studies (MALOVA, P = 0.04). However, the discrimination of genotypes for this tSNP were good and no deviation from HWE was seen in cases suggesting this is a chance occurrence. Therefore we included the data for this tSNP in further analyses.

The comparison of genotype frequencies (P-heterogeneity) between cases and controls in the combined data from all three ovarian cancer studies are presented in Table 1. Table S3 presents the observed genotype frequencies by study. We found no significant differences in genotype frequencies for any of the 12 stSNPs genotyped. The genotypic specific risks in the combined data and the test for trend for each tSNP are also presented in Table 1. We found evidence for a weak protective effect for the rare allele of rs2191249, which was associated with a reduced disease risk in a dose-dependant manner (OR = 0.90 [95%CI 0.82–1.0] P-trend = 0.045) compared with common homozygotes. There was also evidence for a borderline significant effect for the rare allele of rs4988344, which was associated with an increased risk in a dose-dependant manner (OR = 1.15 [95%CI 1.02–1.30], P-trend = 0.02), compared with common homozygotes. There was no heterogeneity between the studies for either of these tSNPs (P = 0.71 and 0.41 respectively). In logistic regression model including both these SNPs there was little attenuation of the per-allele risks (data not shown). There was no difference in the three allele multi-marker tagging haplotype for rs4986765 (P = 0.16) or two allele multi-marker tag for rs1243935 (P = 0.22).

**Figure 1:**
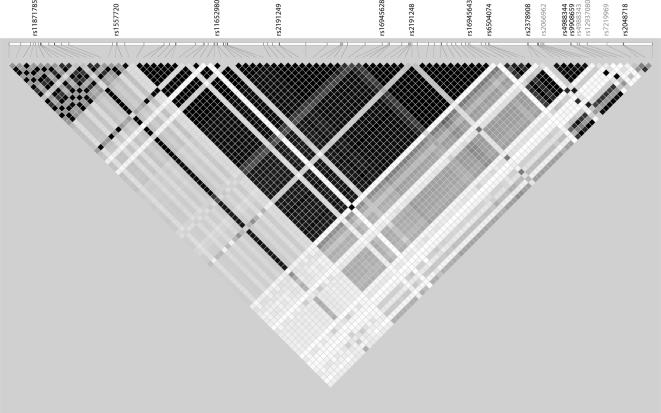
Linkage disequilibrium between the 92 common variants (MAF>0.05) in HapMap CEPH trios. Each square represents the correlation (r^2^) between each pair of SNPs with darker shades representing stronger LD. Tag SNPs are indicated with those SNPs that failed assay design being shown in grey font.

There were two LD blocks with common haplotypes, which accounted for 91% and 83% of all haplotypes respectively (Figure 1). rs11871785, rs1557720, rs11652980, rs2191249, rs16945628 and rs2191248 were situated in block 1 and the remaining tSNPs (rs16945643, rs6504074, rs2378908, rs4988344, rs9908659 and rs2048718) were situated in block 2. The haplotype frequencies for *BRIP1* between cases and controls were estimated after stratification. Haplotype specific risks for individual haplotypes were close to unity compared with the most common one in each block (data not shown). No significant differences in individual haplotype frequencies were seen between cases and controls for breast and ovarian cancer studies, nor in the global test for a haplotype effect (P value for block 1 was 0.68 and 0.43, P value for block 2 was 0.99 and 0.25 respectively for breast and ovarian cancer).

Ovarian cancer is histologically a very heterogeneous disease. Different histological subtypes may show different genetic association, e.g, the proportion of serous tumours is higher in *BRCA1* associated ovarian cancer. Statistical power to identify sub-group effects in the combined series of ovarian cancer cases was limited, so we restricted sub-group analysis to serous cases only (n = 698 in total). The comparison of genotype frequencies (P-heterogeneity) between cases and controls and the genotype specific risks for serous cases in the combined dataset are presented in Table 2. Only rs4988344 showed a marginally significant difference in genotype frequencies between cases and controls (P-het = 0.027). In general, genotype specific risks were similar between serous only cases and all cases combined, although there were slightly increased risks in serous cases for the rare alleles of rs4988344 (P trend = 0.008) and rs2378908 (P-trend = 0.015) (Table 2). For rs2191249, the rare allele protective effect identified in all cases was slightly stronger when serous only cases were considered (P–trend = 0.026).

**Table 2:** Serous type ovarian cancer genotype specific risks for each tSNP

*dbSNP*	# serous cases	# controls	HetOR*[95% CI]	HomOR*[95% CI]	P-het	P_trend
rs11871785	698	4750	1.01 [0.84–1.20]	1.09 [0.84–1.42]	0.80	0.59
rs1557720	698	4229	1.14 [0.94–1.39]	1.16 [0.89–1.51]	0.36	0.20
rs11652980	698	4765	0.97 [0.73–1.28]	1.54 [0.33–7.25]	0.85	0.95
rs2191249	698	4242	0.83 [0.69–1.00]	0.75 [0.51–1.09]	0.08	**0.026**
rs16945628	698	4754	**0.78 [0.65–0.93]**	1.00 [0.77–1.29]	**0.01**	0.21
rs2191248	698	4741	0.94 [0.79–1.12]	1.05 [0.80–1.36]	0.66	0.96
rs16945643	698	4746	1.03 [0.82–1.30]	1.72 [0.72–4.15]	0.50	0.46
rs6504074	698	3968	0.90 [0.74–1.09]	0.93 [0.67–1.30]	0.51	0.35
rs2378908	698	4254	1.21 [0.98–1.49]	1.73 [0.99–3.01]	0.05	**0.015**
rs4988344	698	4265	1.20 [0.98–1.46]	**1.78 [1.10–2.89]**	**0.027**	**0.008**
rs9908659	698	4749	0.87 [0.73–1.04]	0.91 [0.71–1.17]	0.33	0.27
rs2048718	698	4741	0.93 [0.77–1.13]	1.12 [0.89–1.41]	0.22	0.42

* compared with common homozygote. Confidence intervals that do not reach or cross 1.00 and P- values<0.05 are in bold type

## Discussion

The role of abnormal DNA helicase function in the deregulation of DNA repair and genomic stability, and in human cancer development, is well documented. The functional interaction between the DNA helicase *BRIP1* and the breast/ovarian cancer susceptibility gene *BRCA1* makes common variants in *BRIP1* good candidates for low to moderate penetrance susceptibility to both breast and ovarian cancer.

In this study, we evaluated the association between SNPs that efficiently tag the common variation in the *BRIP1* gene and the risks of breast and ovarian cancer using a case-control study design. The tSNPs that we tested were not chosen because they were of any known functional significance. tSNP selection was based on the latest Hapmap data (Phase II release # 20) in CEPH DNA samples, which are of North Western European ancestry. The tSNPs selected using these data provide good power to capture all common variation [25]. Therefore, we are confident that the set of tSNPs we chose adequately tag the known and unknown common variants within the gene.

Previous studies have looked at the association between a handful of common functional *BRIP1* polymorphisms and breast cancer risk. The Ser919Pro variant was found to increase breast cancer risk in families using a ‘kin-cohort’ study design [20]. However, subsequent studies failed to confirm this finding [16], [21]. Ser919Pro (MAF = 0.45) was tagged by rs1557720 (r_p_^2^ = 0.97). We found no evidence of association between this SNP and breast cancer risk (P = 0.39). Our study has 97% power to detect an allele with this frequency with a type I error rate of 0.0001, even if the true relative risk was 1.3.

Another study has indicated that the rare variant Arg173Cys (tSNP rs4988345) impairs protein translocation to the nucleus and might modify breast cancer susceptibility [17], [26]. Since the minor allele for this variant is very rare (MAF = 0.008) in CEPH samples, we have very limited power to confirm or refute this association using the case-control population in the current study.

We found no evidence of association with breast cancer risk for any of the tSNPs analysed in our study. The staged design provides at least 85% power at a Type I error rate of 10^−3^ to detect an allele that explains 0.75% of the excess familial risk of breast cancer and has been tagged with r^2^ = 0.8 – e.g.a co-dominant allele with a frequency of 0.1 that confers a relative risk of 1.27. Therefore, it is unlikely that common variants in *BRIP1* contribute significantly to breast cancer risk. However, we cannot exclude the possibility that the alleles we investigated are associated with smaller risks. Power to detect alleles explaining 0.5% of the excess familial risk is approximately 65%, and power to detect rarer susceptibility variants that are weakly correlated with the polymorphisms we examined is low. Furthermore some known common variants were poorly tagged, because of tSNP assay failure. Again power to detect association with these SNPs is limited.

To our knowledge, this is the first study to evaluate the association between common variants in *BRIP1* and the risks of epithelial ovarian cancer. We found some evidence of association with disease risk for two of the 12 tSNPs tested- rs2191249 and rs4988344. However, these associations were only of borderline significance, and so need to be interpreted with some caution. Despite the large sample size, neither association is highly significant (P = 0.047 and 0.02 respectively) and the P-values have not been adjusted for multiple hypothesis testing. As the tSNPs are correlated, the test statistics are not independent, and standard methods for adjusting for multiple testing, such as the Bonferroni correction, are too conservative. Therefore we used a simulation to determine an empirical P-value for the most significant result (P-trend = 0.02 for rs4988344). In this analysis, we randomly shuffled the case-control status among individuals multiple times, and estimated how frequently a P-value<0.02 was obtained from the randomly permutated data. This method also accounts for the testing of multiple genetic models with each SNP. In 1,000 permutations a P<0.02 was observed on 357 occasions, giving the most significant P-value corrected for multiple testing of 0.36. Thus it is likely that the positive result is a chance finding.

Disease heterogeneity could also lead to false positive reporting or mask the presence of true associations. When we stratified cases according to histological sub-type we found that the strength of association with ovarian cancer risks improved for rs4988344 and rs2378908 when cases of serous histology only were considered (P trend = 0.008 and 0.015 respectively). Once again, caution is required when interpreting these data. There is an inevitable loss of statistical power when stratifying cases into clinical sub-types, and after adjusting for multiple testing, the most significant of these associations (P-trend = 0.008 for rs4988344) was P = 0.17.

Another explanation for a spurious association could be hidden population stratification. This occurs when allele frequencies differ between population sub-groups and cases and controls are drawn differentially from those sub-groups. The three ovarian cancer case-control studies used in the present analysis were from the UK, Denmark and USA. However, all analyses were restricted to subjects of the same ethnic origin (white, Western European) and so population stratification is unlikely to be explanation for erroneous associations. Even if stratification were present, it is unlikely that the same degree of stratification would occur in all three studies.

In combination, the three ovarian cancer studies has more than 80% power to detect a common allele that explains 0.75% of the excess familial risk at a type I error rate of 10^−3^ (for example an allele with frequency 0.2 that confers a relative risk of 1.3), and more than 55% power to detect a common allele that explains 0.5% of the excess familial risk.

Assuming the genetic associations we identified are real, they may either be due to a direct causative effect of the SNPs tested, or because these tSNPs are in linkage disequilibrium and serve as markers for the real determinant of a disease. rs2191249 and rs4988344 are both intronic and neither is strongly correlated with other known SNPs that are more likely to have functional role. The bioinformatics tool PupaSNP (http://pupanp.bioinfo.cnio.es) suggests neither variant has a functional effect or dramatically alters the structure of *BRIP1*. Thus, it seems likely that any true causal variant(s) will be in linkage disequilibrium with rs2191249 or rs4988344. All the known common coding SNPs, 3′UTR SNPs and 5′UTR SNPs were tagged by our selected panel of tSNPs with r^2^>0.95 and were not associated with disease. However, we cannot exclude the possibility that unidentified variants exist in the promoter or regulatory region or intron-exon boundaries, which affect the transcription of *BRIP1,* and are tagged by the two tSNPs for which we find association.

In conclusion, we have genotyped 12 tSNPs that tag the common variants in BRIP1 in breast and ovarian cancer case control series. We found no association with breast cancer risk for any tSNP; but we found evidence of borderline significant associations with invasive ovarian cancer risk for two tSNPs of unknown related function to BRIP1. The observed associations with ovarian cancer risk warrant further evulation in independent case-control studies.

## Supporting Information

Table S1 Primers and probes used for Taqman assays
(0.04 MB DOC)

Table S2 BRIP1 polymorphisms and genotype distributions for the breast cancer case-control study
(0.05 MB DOC)

Table S3 BRIP1 polymorphisms and genotype distributions for the three ovarian cancer case-control studies
(0.15 MB DOC)
