# Supplementary material for: Tagging Single Nucleotide Polymorphisms in the BRIP1 Gene and Susceptibility to Breast and Ovarian Cancer
Source: PLoS One. 2007 Mar 7;2(3):e268. doi: 10.1371/journal.pone.0000268 (PMC1800910; doi:10.1371/journal.pone.0000268)
Supplement: Table S1 — Primers and probes used for Taqman assays (0.04 MB DOC) [file pone.0000268.s001.doc]

**Table S1:** Primers and probes used for Taqman^TM^ assays

| SNP | Forward primer (5’-3’) | Reverse primer (5’-3’) | Vic-probe^1^ | Fam-probe^1^ | Annealing temperature^2^ |
| --- | --- | --- | --- | --- | --- |
| rs11871785 | tcatcttggaacagaatattaactctgaaagaaa | tccctgctgtggaatttgatagttt | CCTAGCCCAAGTTATAA | CTAGCCCAGGTTATAA | 60°C |
| rs1557720 | ccctcacccaggtttacttctg | gctgtgcctggtgttgaagaata | AACAACAGAGACTCTG | AACAACAGAGTCTCTG | 60°C 50 cycles |
| rs11652980 | gctttggtatgagaccctaactga | ctgaccactgagctgacttattga | CTCCCCCTAGGATACA | CTCCCCCTACGATACA | 60°C |
| rs2191249 | actagattgtgaagcgatgctttct | gccatctgaatctgctgtaaatttgt | ACTTGAAAGAAATGTTTTGG | ACTTGAAAGAAATTTTTTGG | 60°C |
| rs16945628 | ggcatagaagaaggcataagtagaca | ctggaaaatacagaatggcacaaagaa | CTCAAAGGTGAACATT | ACTCAAAGATGAACATT | 60°C |
| rs2191248 | gcctttatatgaaaaatatgtttgattatgccaga | gcttaactggcaaggaacaattca | TTAACTAGGCTTCTTGAGAAA | ACTAGGCTTCTTGGGAAA | 60°C |
| rs16945643 | cagtttaagaacaagcatatcataaagatgactg | gagactcattgctataatcacatctatgtaaagt | ACCTGCTATAAAGCAAAA | CTGCTATGAAGCAAAA | 60°C |
| rs6504074 | gcccttcctcctccctctt | ctgtccagttccctttcttcctt | CTGGATCTATTAGTACGACAAA | TGGATCTATTAGTAAGACAAA | 60°C 50 cycles |
| rs2378908 | ccacgctcggcccaata | gtaaacatttccaccatagagaacactact | ACGATTTTTTTGAGAGCCT | ATTACGATTTTTTTAAGAGCCT | 60°C |
| rs4988344 | gagctgttttggcctttgagaac | ccaaagcaatgacgttttctaatctgt | ACTTCTAGTTCACCTTAAAC | TTCTAGTTCACGTTAAAC | 60°C |
| rs9908659 | agtgatgtatcaaaattagagtattgtaaaatgaacttct | ggactttccaatattgctatatttgatgtcc | ATGGGACTGTTTTATG | ATGGGACTATTTTATG | 60°C |
| rs2048718 | gcccttcctcctccctctt | gtgggtcgaggaaaggtaacg | CCCCAGTCCTGCACAC | CCCCAATCCTGCACAC | 60°C |

^1^ variable nucleotide underlined, ^2^ all assay performed 40 PCR cycles except specified
