# Supplementary material for: Tagging Single Nucleotide Polymorphisms in the BRIP1 Gene and Susceptibility to Breast and Ovarian Cancer
Source: PLoS One. 2007 Mar 7;2(3):e268. doi: 10.1371/journal.pone.0000268 (PMC1800910; doi:10.1371/journal.pone.0000268)
Supplement: Table S2 — BRIP1 polymorphisms and genotype distributions for the breast cancer case-control study (0.05 MB DOC) [file pone.0000268.s002.doc]

**Table S2: *BRIP1*** polymorphisms and genotype distributions for the breast cancer case-control study

| **dbsnp** | **Controls** | | | |  | **Cases** | | | |  | **Control** | |
| --- | --- | --- | --- | --- | --- | --- | --- | --- | --- | --- | --- | --- |
|  | **AA**^1^ | **Aa^2^** | **aa^3^** | Total |  | **AA^1^** | **Aa^2^** | **aa^3^** | Total |  | **MAF** | **HWE-P** |
| rs11871785 | 958 | 1062 | 247 | 2267 |  | 926 | 991 | 264 | 2181 |  | 0.34 | 0.06 |
| rs1557720 | 803 | 1066 | 378 | 2247 |  | 793 | 1017 | 346 | 2156 |  | 0.41 | 0.45 |
| rs11652980 | 2004 | 265 | 6 | 2275 |  | 1931 | 247 | 4 | 2182 |  | 0.06 | 0.37 |
| rs2191249 | 1239 | 882 | 156 | 2277 |  | 1193 | 825 | 171 | 2189 |  | 0.26 | 0.95 |
| rs16945628 | 1012 | 1008 | 251 | 2271 |  | 975 | 938 | 263 | 2176 |  | 0.33 | 1.00 |
| rs2191248 | 980 | 995 | 289 | 2264 |  | 912 | 976 | 274 | 2162 |  | 0.35 | 0.14 |
| rs16945643 | 1958 | 292 | 15 | 2265 |  | 1872 | 284 | 15 | 2171 |  | 0.07 | 0.26 |
| rs2378908 | 1735 | 497 | 45 | 2277 |  | 1663 | 495 | 32 | 2190 |  | 0.13 | 0.18 |
| rs4988344 | 1609 | 616 | 53 | 2278 |  | 1552 | 585 | 52 | 2189 |  | 0.16 | 0.51 |
| rs9908659 | 892 | 1047 | 327 | 2266 |  | 858 | 979 | 327 | 2164 |  | 0.38 | 0.49 |
| rs2048718 | 655 | 1151 | 458 | 2264 |  | 655 | 1063 | 452 | 2170 |  | 0.46 | 0.24 |

1: Common homozygous, 2: heterozygous, 3: rare homozygous, 4: Comparison of genotype frequencies in cases and controls [chisq 2d.f]
