# Supplementary material for: Tagging Single Nucleotide Polymorphisms in the BRIP1 Gene and Susceptibility to Breast and Ovarian Cancer
Source: PLoS One. 2007 Mar 7;2(3):e268. doi: 10.1371/journal.pone.0000268 (PMC1800910; doi:10.1371/journal.pone.0000268)
Supplement: Table S3 — BRIP1 polymorphisms and genotype distributions for the three ovarian cancer case-control studies (0.15 MB DOC) [file pone.0000268.s003.doc]

**Table S3: *BRIP1*** polymorphisms and genotype distributions for the three ovarian cancer case-control studies

| **dbsnp** | **Study** | **Controls** | | | |  | **Cases** | | | |  | **Control** | |
| --- | --- | --- | --- | --- | --- | --- | --- | --- | --- | --- | --- | --- | --- |
|  |  | **AA**^1^ | **Aa^2^** | **aa^3^** | Total |  | **AA^1^** | **Aa^2^** | **aa^3^** | Total |  | **MAF** | **HWE-P** |
| rs11871785 | SEARCH | 1302 | 1455 | 353 | 3110 |  | 279 | 350 | 98 | 727 |  | 0.35 | 0.08 |
|  | STAN | 165 | 191 | 67 | 423 |  | 131 | 151 | 39 | 321 |  | 0.38 | 0.35 |
|  | MALOVA | 535 | 538 | 144 | 1217 |  | 195 | 203 | 46 | 444 |  | 0.34 | 0.62 |
|  | Combined | 2002 | 2184 | 564 | 4750 |  | 605 | 704 | 183 | 1492 |  | 0.35 |  |
| rs1557720 | SEARCH | 1115 | 1468 | 512 | 3095 |  | 262 | 359 | 110 | 731 |  | 0.40 | 0.44 |
|  | STAN | 175 | 193 | 56 | 424 |  | 121 | 140 | 63 | 324 |  | 0.36 | 0.81 |
|  | MALOVA | 245 | 347 | 118 | 710 |  | 89 | 136 | 52 | 277 |  | 0.41 | 0.79 |
|  | Combined | 1535 | 2008 | 686 | 4229 |  | 472 | 635 | 225 | 1332 |  | 0.40 |  |
| rs11652980 | SEARCH | 2772 | 341 | 10 | 3123 |  | 645 | 80 | 1 | 726 |  | 0.06 | 0.89 |
|  | STAN | 374 | 49 | 0 | 423 |  | 294 | 25 | 2 | 321 |  | 0.06 | 0.24 |
|  | MALOVA | 1120 | 98 | 1 | 1219 |  | 404 | 38 | 1 | 443 |  | 0.04 | 0.44 |
|  | Combined | 4266 | 488 | 11 | 4765 |  | 1343 | 143 | 4 | 1490 |  | 0.05 |  |
| rs2191249 | SEARCH | 1701 | 1196 | 218 | 3115 |  | 419 | 275 | 37 | 731 |  | 0.26 | 0.69 |
|  | STAN | 239 | 155 | 33 | 427 |  | 192 | 109 | 20 | 321 |  | 0.26 | 0.27 |
|  | MALOVA | 398 | 251 | 51 | 700 |  | 158 | 99 | 19 | 276 |  | 0.25 | 0.19 |
|  | Combined | 2338 | 1602 | 302 | 4242 |  | 769 | 483 | 76 | 1328 |  | 0.26 |  |
| rs16945628 | SEARCH | 1389 | 1375 | 356 | 3120 |  | 353 | 310 | 68 | 731 |  | 0.33 | 0.57 |
|  | STAN | 191 | 177 | 55 | 423 |  | 148 | 141 | 34 | 323 |  | 0.34 | 0.17 |
|  | MALOVA | 534 | 546 | 131 | 1211 |  | 195 | 186 | 60 | 441 |  | 0.33 | 0.63 |
|  | Combined | 2114 | 2098 | 542 | 4754 |  | 696 | 637 | 162 | 1495 |  | 0.33 |  |
| rs2191248 | SEARCH | 1357 | 1386 | 367 | 3110 |  | 297 | 325 | 104 | 726 |  | 0.34 | 0.65 |
|  | STAN | 202 | 174 | 45 | 421 |  | 139 | 142 | 41 | 322 |  | 0.31 | 0.41 |
|  | MALOVA | 453 | 602 | 155 | 1210 |  | 183 | 205 | 47 | 435 |  | 0.38 | 0.04 |
|  | Combined | 2012 | 2162 | 567 | 4741 |  | 619 | 672 | 192 | 1483 |  | 0.35 |  |
| rs16945643 | SEARCH | 2670 | 424 | 17 | 3111 |  | 629 | 93 | 2 | 724 |  | 0.07 | 0.97 |
|  | STAN | 357 | 62 | 4 | 423 |  | 268 | 47 | 6 | 321 |  | 0.08 | 0.48 |
|  | MALOVA | 1019 | 189 | 4 | 1212 |  | 360 | 77 | 3 | 440 |  | 0.08 | 0.12 |
|  | Combined | 4046 | 675 | 25 | 4746 |  | 1257 | 217 | 11 | 1485 |  | 0.08 |  |
| rs6504074* | SEARCH | 457 | 325 | 65 | 847 |  | 417 | 270 | 38 | 725 |  | 0.27 | 0.50 |
|  | STAN | 225 | 156 | 40 | 421 |  | 170 | 117 | 37 | 324 |  | 0.28 | 0.09 |
|  | MALOVA | 340 | 258 | 52 | 650 |  | 137 | 104 | 19 | 260 |  | 0.28 | 0.76 |
|  | Combined | 2150 | 1499 | 319 | 3968 |  | 724 | 491 | 94 | 1309 |  | 0.27 |  |
| rs2378908 | SEARCH | 2357 | 709 | 58 | 3124 |  | 521 | 170 | 16 | 707 |  | 0.13 | 0.58 |
|  | STAN | 318 | 91 | 10 | 419 |  | 243 | 70 | 7 | 320 |  | 0.13 | 0.26 |
|  | MALOVA | 564 | 137 | 10 | 711 |  | 199 | 73 | 3 | 275 |  | 0.11 | 0.61 |
|  | Combined | 3239 | 937 | 78 | 4254 |  | 963 | 313 | 26 | 1302 |  | 0.13 |  |
| rs4988344 | SEARCH | 2198 | 855 | 73 | 3126 |  | 498 | 203 | 28 | 729 |  | 0.16 | 0.34 |
|  | STAN | 309 | 103 | 15 | 427 |  | 228 | 86 | 9 | 323 |  | 0.16 | 0.09 |
|  | MALOVA | 526 | 174 | 12 | 712 |  | 188 | 82 | 8 | 278 |  | 0.14 | 0.58 |
|  | Combined | 3033 | 1132 | 100 | 4265 |  | 914 | 371 | 45 | 1330 |  | 0.16 |  |
| rs9908659 | SEARCH | 1230 | 1446 | 439 | 3115 |  | 275 | 339 | 117 | 731 |  | 0.37 | 0.67 |
|  | STAN | 191 | 172 | 59 | 422 |  | 132 | 147 | 43 | 322 |  | 0.34 | 0.05 |
|  | MALOVA | 405 | 615 | 192 | 1212 |  | 169 | 203 | 67 | 439 |  | 0.41 | 0.10 |
|  | Combined | 1826 | 2233 | 690 | 4749 |  | 576 | 689 | 227 | 1492 |  | 0.38 |  |
| rs2048718 | SEARCH | 901 | 1576 | 634 | 3111 |  | 236 | 341 | 145 | 722 |  | 0.46 | 0.25 |
|  | STAN | 131 | 195 | 95 | 421 |  | 91 | 157 | 74 | 322 |  | 0.46 | 0.17 |
|  | MALOVA | 352 | 632 | 225 | 1209 |  | 118 | 215 | 96 | 429 |  | 0.45 | 0.05 |
|  | Combined | 1384 | 2403 | 954 | 4741 |  | 445 | 713 | 315 | 1473 |  | 0.46 |  |

1: Common homozygous, 2: heterozygous, 3: rare homozygous, 4: Comparison of genotype frequencies in cases and controls [chisq 2d.f], * rs6504074 did not include breast cancer controls.
